# *Bergbambos* and *Oldeania*, new genera of African bamboos (Poaceae, Bambusoideae)

**DOI:** 10.3897/phytokeys.25.6026

**Published:** 2013-08-23

**Authors:** Chris M. A. Stapleton

**Affiliations:** 1 11 Chandos Close, Amersham, HP6 6PJ, United Kingdom

**Keywords:** *Thamnocalamus*, *Yushania*, *Arundinarieae*, new genus, Africa

## Abstract

Two new monotypic genera, *Bergbambos* and *Oldeania* are described for African temperate bamboo species in the tribe *Arundinarieae*, after a comparison of their morphological characteristics with those of similar species from Asia. Morphological differences are supported by their isolated geographical distributions. Molecular evidence does not support the inclusion of these species in related Asian genera, recognising them instead as distinct lineages. New combinations *Bergbambos tessellata* and *Oldeania alpina* are made.

## Introduction

While Asian temperate bamboos have received critical attention over recent decades (Stapleton 1994, Wong 1995, Li et al. 2006), the generic placement of the temperate bamboos of Africa has not been properly addressed. There seem to be only two temperate bamboo species on the African mainland, currently enumerated most frequently as *Thamnocalamus tessellatus* (Nees) Soderstrom & R.P. Ellisand *Yushania alpina* (K. Schum.) W.C. Lin. These species are in tribe Arundinarieae Nees ex Asch. & Graebn., a group also known as the northern temperate clade, identified as a strongly supported monophyletic group from the first molecular analyses of bamboos onwards (Watanabe et al. 1994, Zhang 1996).

Tribe Arundinarieae contains woody bamboos with semelauctant synflorescences (lacking a capability for indeterminate growth from buds subtended by the basal spikelet bracts), ebracteate or partially bracteate synflorescence paraclades (reduced sheathing subtending inflorescence branches) and 3 stamens in each floret. They constitute ca. 800 of the ca. 1400 woody bamboos, and are found in Asia, Africa, and the USA, having a montane or subtropical to temperate distribution.

Molecular studies reviewed by Bamboo Phylogeny Group (2012) suggest that semelauctant inflorescences with 3 stamens and reduced branch sheathing have evolved from tropical bamboos at least twice, once to give the northern temperate clade *Arundinarieae* of Asia and Africa, spreading to N America, and on separate occasions in Central & South America within the *Bambuseae* Kunth ex Dumort., principally to give *Chusqueinae* Bews, with these characters also evolving on a smaller scale within the *Arthrostylidiinae* Bews and *Guaduinae* Soderstr. & R. P. Ellis as well.

Most of the older 3-stamened species were placed at some time in *Arundinaria* Michx., which has 529 combinations, but that genus is now widely recognised as containing only 3 species, all from the Southeast USA (Stapleton et al. 2004, Zeng et al. 2010, Bamboo Phylogeny Group 2012). Treatments of the other species of tribe Arundinarieae vary, according to the breadth of generic concept used, and which characters are given greatest weight. For example, the group of Asian species morphologically closest to *Arundinaria* could be placed (Zhang et al. 2012) either in a polyphyletic broad interpretation of *Arundinaria* (e.g. Li et al. 2006), in a polyphyletic broad interpretation of *Bashania* Keng f. & T.P. Yi (e.g. Keng and Yi 1996), or in the monophyletic *Sarocalamus* Stapleton (Stapleton et al. 2004, Bamboo Phylogeny Group 2012). The morphologically more distinct species are currently placed in other genera, 27 of which were recognised by Bamboo Phylogeny Group (2012), out of a total of 42 genera that have been described within the tribe.

There appears to have been a rapid and relatively recent diversification within bamboos with 3 stamens, including tribe Arundinarieae (Stapleton et al. 2009, Hodkinson et al. 2010, Zhang et al. 2011, Kelchner and BPG 2013), especially those found in montane and temperate areas such as the Andes, the Himalayas, and Northeast Asia. There have also been several reports of hybridisation, reviewed by Triplett et al. (2010) and Zhang et al. (2012). Hybridisation may well have been common in the bamboos, as mechanisms to avoid it have not been documented. Recent rapid diversification and hybridisation, combined with long generation times, appear to have limited the ability of DNA analyses to resolve phylogenetic patterns and define well supported groups for taxonomy, especially at the generic level (Stapleton et al. 2009, Hodkinson et al. 2010), despite reasonable or sometimes very substantial morphological variation.

In the absence of reliable molecular analyses, for the purpose of descriptive treatments of bamboo species (Li et al. 2006, Wong 1995, Dransfield 2000, Widjaja 1997) a more traditional morpho-geographic approach has been maintained in the classification of Asian bamboos. It has only been possible to use molecular data for the elimination of blatantly polyphyletic groups, rather than the determination of monophyletic ones. Attempts to group the genera substantially (e.g. Clayton and Renvoize 1986, Chao and Renvoize 1989) have resulted in polyphyletic and paraphyletic groups, or clades with weak support that are inconsistent in different analyses.

Rapid recent diversification seems to have spawned a host of small groups, often distinguished by relatively minor characters. Combining them together into a few large genera has not been possible without establishing excessively variable genera that are difficult to define and demonstrably polyphyletic. On the other hand recognising only half of the genera described would still lead to a generic concept that is unusually narrow in the grasses. The latter procedure has been followed (Bamboo Phylogeny Group 2012), largely because it has been found unavoidable if a functional binomial classification system is to be maintained. This is necessary in order to allow pragmatic field identification, and subsequently improve sustainable utilisation and conservation of these species, many of which have a limited range of distribution and are threatened by changes in land use and climate. A substantial proportion of the woody bamboos are yet to be described, and the lack of a functional and stable nomenclatural system for field identification has been a major factor preventing their recognition.

**Map 1.**
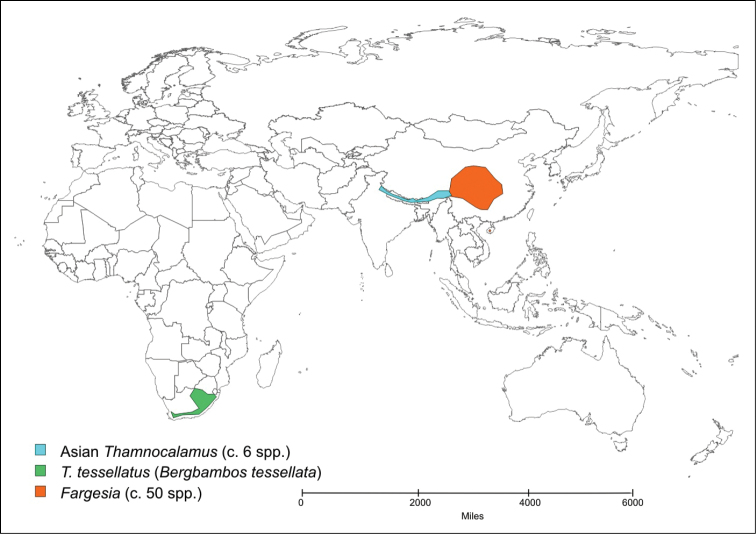
Distribution of *Thamnocalamus tessellatus*, *Thamnocalamus* in Asia, and *Fargesia*.

**Map 2.**
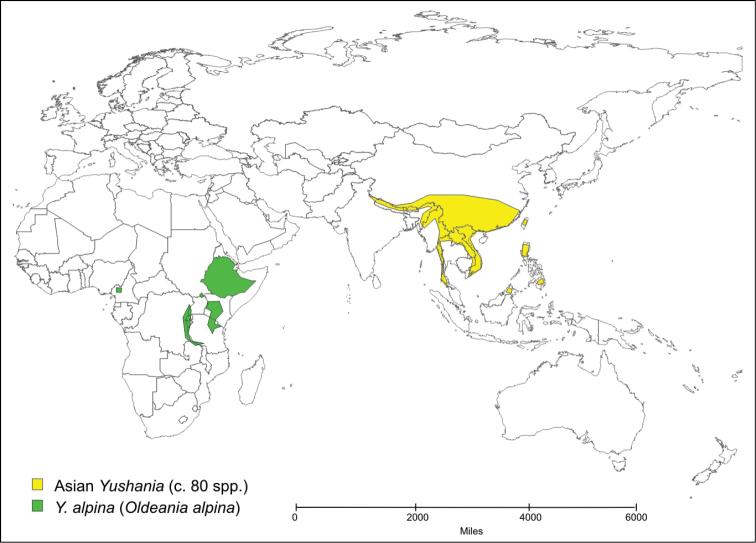
Distribution of *Yushania alpina* and Asian *Yushania* species.

Only two species of temperate bamboo have been described from the African mainland. *Thamnocalamus tessellatus* (Nees) Soderstrom & R.P. Ellis is from mountains in southern Africa, while *Yushania alpina* (K. Schum.) W.C. Lin is from mountains in several countries across tropical Africa. *Yushania alpina* was described initially in *Arundinaria*, and *Thamnocalamus tessellatus* was soon transferred into that genus from *Nastus* Juss. They were more recently moved into the morphologically closer Asian genera, *Thamnocalamus* Munroand *Yushania* Keng f., the geographically closest representatives of which are found in the Western Himalayas, Map 1 and Map 2.

Three further, less well known species, *Thamnocalamus ibityensis* (A. Camus) Ohrnb., *Yushania madagascariensis* (A. Camus) Ohrnb. and *Yushania humbertii* (A. Camus) Ohrnb. (including *Yushania ambositrensis* (A. Camus) Ohrnb.) were described from Madagascar. *Thamnocalamus ibityensis* has been considered conspecific with *Thamnocalamus tessellatus* (Chao & Renvoize, 1989), but it would appear to have substantially different branch sheathing. The two *Yushania* species would appear to share characteristics with *Yushania alpina*, but their culms, branching and culm sheaths are not known. *Yushania ambositrensis* resolved in a clade with *Yushania alpina* (Triplett, 2008), but it is not clear how closely related they really are to *Yushania alpina*, or to each other, and which species names should be recognised. Further field work on temperate species of Madagascar is required, as existing collections are incomplete, although any such species may have already become extinct.

## Comparison of morphological characters

Systematics within the grass family has traditionally given greater weight to floral than to vegetative characters. This has often led to polyphyletic genera in the bamboos, the superficiality of their similarities and their separate origins only being revealed by in-depth morphological investigations and/or molecular studies. In order to allow deeper, more objective morphological comparisons and to allow inclusion of consistent and accurate vegetative as well as floral characters in descriptions, the morphology of woody bamboos has been reviewed in depth (Stapleton 1997, available online). Recent bamboo treatments (Judziewicz et al. 1999, Li et al. 2006, Triplett et al. 2006, BPG 2012) have employed these revised concepts and terminologies, and they are followed here.

The characters and character states considered important at the generic level for distinguishing the two African species from similar Asian genera are given in Table 1.

**Table 1.** Principal morphological characters of *Bergbambos*, *Oldeania*, and Asian members of 5 similar genera.

	*Bergbambos* (*Thamnocalamus tessellatus*)	*Thamnocalamus*	*Fargesia*	*Oldeania* (*Yushania alpina*)	*Yushania*	*Borinda*	*Chimonocalamus*
synflorescence	raceme, not unilateral	raceme to panicle, not unilateral	raceme, unilateral	panicle, not unilateral	panicle, not unilateral	panicle, not unilateral	panicle, not unilateral
paraclades	largely ebracteate	substantially bracteate	variably bracteate	largely ebracteate	largely ebracteate	largely ebracteate	largely ebracteate
pedicel	scabrous	glabrous	glabrous	glabrous	glabrous	glabrous	glabrous
fertile florets	1	2–several	2–several	2–several	2–several	2–several	2–several
glume bud remnants	absent	variable	present	absent	variable	variable	absent
rhizomes	short-necked	short-necked	short-necked	long-necked	long-necked	short-necked	short-necked
clump form	unicaespitose	unicaespitose	unicaespitose	culms solitary	pluricaespitose	unicaespitose	unicaespitose
nodes	without roots	without roots	without roots	with short roots	without roots	without roots	with root thorns
supranodal ridge	obscure	obscure	obscure	well developed	obscure	obscure	well developed
culm internodes	terete	terete	terete	sulcate	terete	terete	terete
branch sheathing	reduced	complete	reduced	reduced	reduced	reduced	complete
branch orientation	erect	erect	erect	spreading	erect to spreading	erect	spreading
mid-culm branches	5–7	3–8	5–7	3–7	5–11	5–7	3
culm sheath blades	erect or reflexed	usually erect	erect or reflexed	usually reflexed	erect or reflexed	erect or reflexed	usually reflexed

### Thamnocalamus tessellatus

Previous generic placements of *Thamnocalamus tessellatus* were based upon an incomplete knowledge of its morphology. *Nastus tessellatus* Nees was described before its flowers were known, and transferred into *Arundinaria* (Munro 1868) simply as it bore “very great resemblance” to that genus. Later discovery of its flowers has shown that it indeed has 3 stamens, rather than the 6 of *Nastus*, but it has pachymorph rhizomes (see Stapleton 1997: fig. 1) rather than the leptomorph rhizomes of *Arundinaria*.

It was transferred into *Thamnocalamus* largely on the basis of leaf anatomical characters by Soderstrom and Ellis (1982), who found that *Arundinaria tessellata* shared 10 characters out of 11 with *Thamnocalamus spathiflorus* (Trin) Munro, while it only shared 7 characters with *Fargesia nitida* (Mitford) Keng f. However, *Arundinaria tessellata* also shared only 5 characters with *Thamnocalamus aristatus* E.G. Camus, while the possibly conspecific *Thamnocalamus spathiflorus* and *Thamnocalamus aristatus* themselves only shared 6 out of 11 characters. When morphological characters other than those of leaf anatomy, along with more recent molecular results are taken into account, it would appear that the anatomical characters used by Soderstrom and Ellis (1982) are more informative at the level of species or below rather than at generic level.

**Figure 1.**
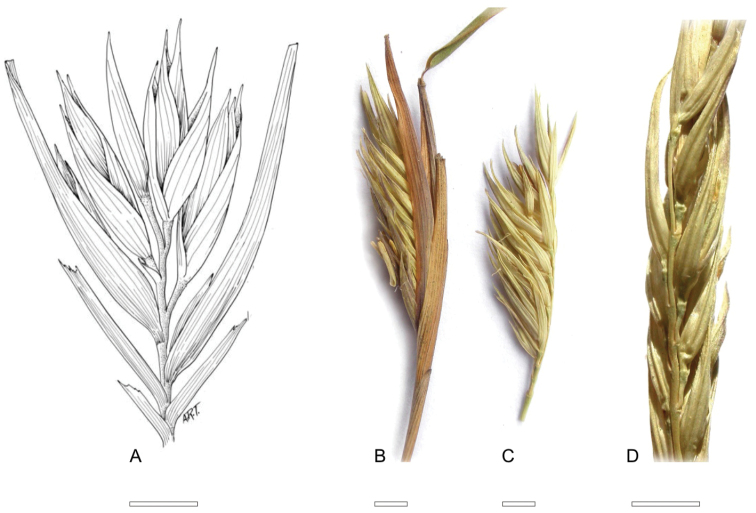
Raceme of *Thamnocalamus tessellatus* (**A**), compared to: **B** *Fargesia nitida*, lateral view with enclosing sheaths; **C** *Fargesia nitida* with enclosing sheaths removed **D** *Fargesia nitida*, dorsal view, sheaths removed. **A** from Soderstrom and Ellis (1982), drawn by A. R. Tangerini, © Smithsonian Institution. **B, C, D** from Stapleton 1061b. Scale bars 2 mm.

The synflorescence of *Thamnocalamus tessellatus* has been well illustrated in Hooker’s Icones Plantarum (Prain 1913: Tab 2930 http://www.botanicus.org/page/1349516), and by Soderstrom and Ellis (1982). When examined closely, it can be seen that the synflorescence of *Thamnocalamus tessellatus* has similarities to those of both *Thamnocalamus* and *Fargesia* Franchet, see Table 1, as they are compressed, and are associated with several supporting sheaths. However, while *Thamnocalamus* has loose racemose panicles, *Thamnocalamus tessellatus*, like *Fargesia*, consistently bears short racemes. These are structurally very similar to those of *Fargesia*, but differences arise in the arrangement of the florets. In *Fargesia* the racemes are held tightly within imbricating sheaths, which can extend well beyond the spikelets. Development within the sheaths forces them to emerge to one side and appear unilateral, with the pedicels tightly pressed against the rhachis. Those of *Thamnocalamus tessellatus* are more cylindrical, the spikelets not so constricted by the sheaths, and the pedicels are free to develop in a normal distichous fashion, Figure 1.

In addition, in *Thamnocalamus tessellatus* the pedicels are scabrous, the glumes of each spikelet are basally tight and contain no vestigial bud remnants, and the racemes are usually largely ebracteate. The usually single fertile florets also distinguish *Thamnocalamus tessellatus* from other *Thamnocalamus* and *Fargesia* species, but this character should be treated with caution as it can be a specific as well as a generic character.

**Figure 2.**
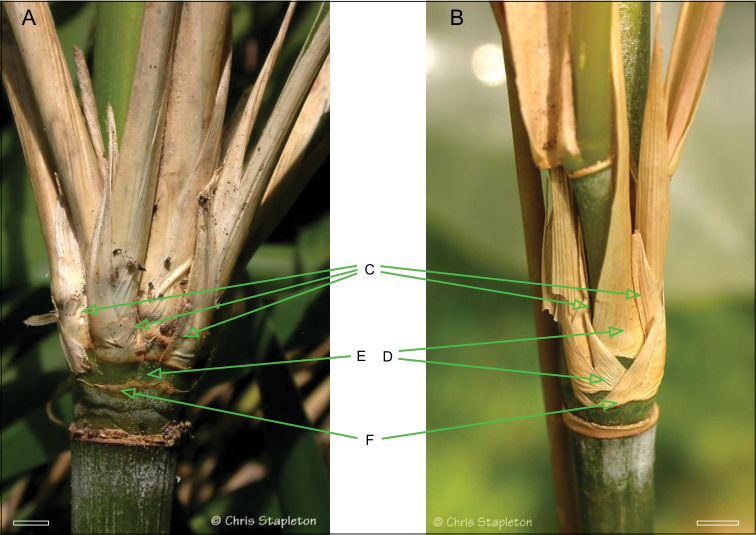
Comparison of branch complement sheathing from mid-culm nodes of *Thamnocalamus tessellatus* (**A**) and *Thamnocalamus crassinodus* (**B**) **C** Lateral branch prophylls **D** Sheaths obscuring prophyll bases in *Thamnocalamus crassinodus* **E** Equivalent sheaths completely absent in *Thamnocalamus tessellatus*, prophylls visible **F** Branch bud prophyll, removed in **A**, still present in **B**. Scale bars 1 cm. From http://www.bamboo-identification.co.uk

*Thamnocalamus tessellatus* also hasvegetative characteristics that distinguish it, notably from Asian members of *Thamnocalamus*, (see Table 1). A close inspection of the branching reveals not the pattern seen in species such as *Thamnocalamus crassinodus* (T.P. Yi) Demoly, but instead the substantial reduction in sheathing seen in *Fargesia*, *Yushania*, and *Borinda* Stapleton, Figure 2.

The branches of *Thamnocalamus tessellatus* are subequal, arranged side by side through strong compression of the basal internodes of the central branch, accompanied by loss of some of the sheaths at the nodes, Fig. 2A, cf *Thamnocalamus crassinodus*, Fig. 2B. This allows lateral branch prophylls to be seen side by side without any intervening sheaths. These patterns were contrasted by Stapleton (1991; 1994: fig. 1; 1997: fig. 2), and also illustrated for *Thamnocalamus tessellatus* by Soderstrom and Ellis (1982: fig.1, fig. 4).

**Figure 3.**
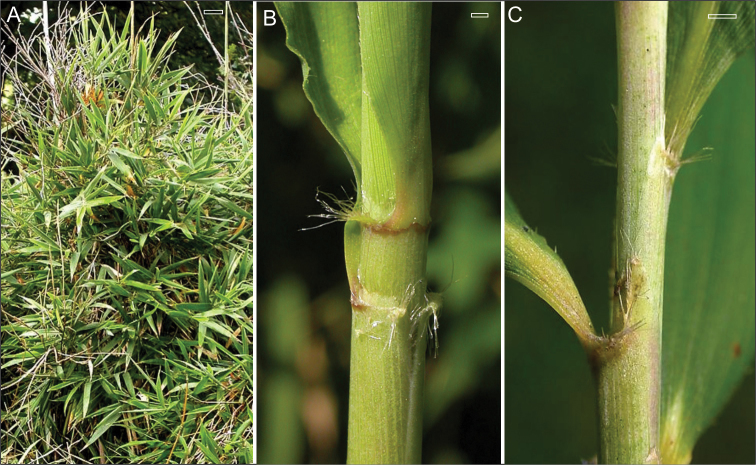
*Thamnocalamus tessellatus*. **A** Random orientation of leaf blade; **B** and **C** Irregular orientation of delicate oral setae. Scale bars **A** 10 cm, **B** and **C** 2 mm. From http://www.bamboo-identification.co.uk/html/tessellatus.html

In addition to the synflorescence and branching, *Thamnocalamus tessellatus* also differs in minor details that are harder to quantify, including the more varied orientation of the foliage leaves, and the delicate appearance of its oral setae and their more varied orientation, Figure 3.

Thus in terms of vegetative macro-morphological characteristics important at the generic level, *Thamnocalamus tessellatus* is closer to *Fargesia* than to *Thamnocalamus*, but can be distinguished from both. In general appearance it resembles a coastal species of *Pleioblastus* Nakai from Japan, with rather loose clumps, erect culms with short branches bearing coarse, irregularly arranged foliage with persistent sheaths. This contrasts with the delicate foliage leaves, all oriented towards the light on pendulous branches seen in Himalayan speciesof *Thamnocalamus* and in *Fargesia*. This is likely to be associated with the more open ecological habitat in which *Thamnocalamus tessellatus* is found, rather than the darker forest understorey habitats of Asian *Thamnocalamus* and *Fargesia* species.

### Yushania alpina

The synflorescence of *Yushania alpina* is practically indistinguishable from those of several Asian and American bamboos, including species of *Arundinaria*, *Sarocalamus*, and *Yushania*—an open panicle with nearly complete reduction in sheathing at points of branching so that it is essentially ebracteate. However, the sheaths are often reduced to small tough bracts, as well as the more delicate sheath remnants or tufts of hairs seen in similar genera. In addition the lateral spikelets are more often sessile or subsessile, without a long pedicel. However, these characters are relatively minor and quite variable.

**Figure 4.**
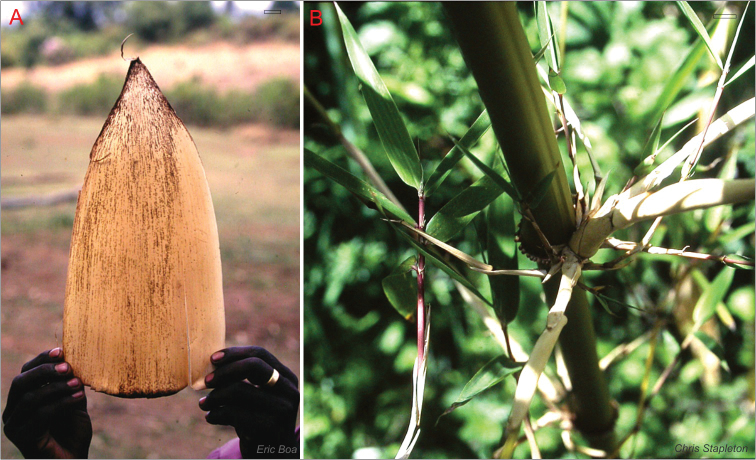
*Yushania alpina*. **A** culm sheath **B** leaf sheaths, culm node with ring of thorn-like aerial roots and distinct supra-nodal ridge, sulcate internode, and dominant central branch. Scale bars top right, 2 cm.

*Yushania alpina* is more distinct vegetatively. Reaching heights of up to 20m in its natural habitat, the tall, very erect culms are potentially much larger than those of any Asian species of *Yushania*, which only reach a maximum height of about 7m. Culm nodes and branching also differ substantially from those of Asian species of *Yushania*, Figure 4.

**Figure 5.**
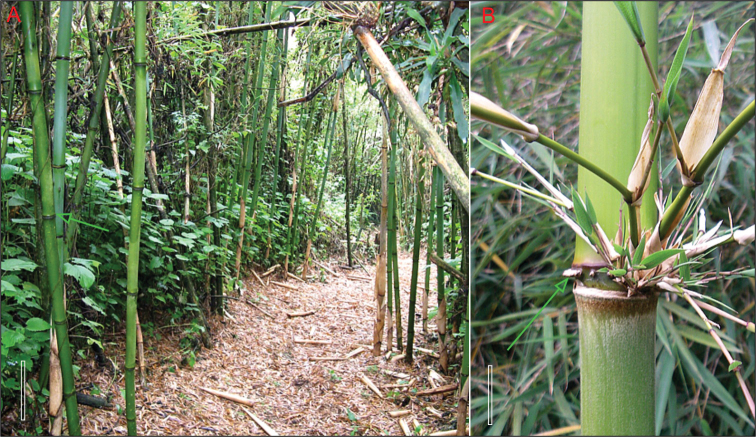
*Yushania alpina* **A** tall culms arising separately with prominent supra-nodal ridges (arrowed), Rwenzori, Uganda **B** culm node with ring of thorn-like aerial roots (arrowed), Mt. Kenya.Scale bars **A** 25 cm, **B** 5 cm. Photos courtesy of: (**A**) Peter Gill, (**B**) Harry Jans, www.jansalpines.com

Branches vary in size more than those of Asian *Yushania* species. The central branch is strongly dominant, and the first two lateral branches are also strong. The orientation of the branches is less erect than those of most species of *Yushania*, becoming nearly horizontal. Above the branches the internode is distinctly sulcate, much more prominently than is seen in Asian *Yushania* species, as a result of the development of strong branches. Moreover there is often a dense ring of short, partially developed aerial roots at nodes in the lower part of the culm, often extending into the mid-culm region as well. This character is only known in species of *Chimonocalamus* Hsueh & T.P. Yi, and the leptomorph-rhizomed *Chimonobambusa* Makino among the Asian temperate bamboos. The roots are not as sharp and thorn-like as those seen in *Chimonocalamus* and *Chimonobambusa*, but they can be very distinct and prominent. Nodes have a distinct infranode between the culm sheath attachment and the supranodal ridge, which is well developed, Figure 5.

In its natural habitat, the open stands have a widely spaced appearance closer to that of a species of *Phyllostachys* Siebold & Zucc., rather than the denser thickets of Asian *Yushania* species, because the rhizomes have consistently long necks, giving solitary culms rather than the denser clusters of pluricaespitose culms seen in Asian species of *Yushania*.

Branch structure and sheathing is difficult to distinguish from that of *Yushania* or *Fargesia*. Although the prophyll is usually 2-keeled, there is replication side by side of lateral branch initials without intervening sheaths. In this way it differs fundamentally from *Chimonocalamus*, which has only 3 branches and full sheathing.

## Discussion

The morphological differences between *Thamnocalamus tessellatus*, *Yushania alpina* and other representatives of these and similar Asian genera suggest that although the two African bamboos share several characters and presumably common ancestors with Asian bamboos, they are not as closely related to their Asian relatives as previously thought.

The morphological distinctions are supported by geographical isolation, (see Maps 1 and 2). Long-distance dispersal of temperate bamboos is highly unlikely because of a lack of any specialized seed dispersal mechanism or dormancy, brief viability of seed, exacting habitat requirements, and extremely infrequent flowering (Stapleton et al. 2004).

Together the morphological distinctions and geographical isolation justify the recognition of two new genera, following the existing relatively narrow generic concepts applied in the northern temperate clade, tribe *Arundinarieae*.

The new genera are keyed out below along with their 6 morphologically closest relatives in the tribe including the two Asian genera with distinct nodal thorns, as well as the North American type genus of the tribe, *Arundinaria*, and its Asian analogue, *Sarocalamus*.

### Key to *Bergbambos*, *Oldeania* and related genera

**Table:** 

1	Rhizome leptomorph	2
–	Rhizome pachymorph	4
2	Basal culm nodes with thorns, branches spreading	*Chimonobambusa*
–	Basal culm nodes without thorns, branches erect	3
3	Pedicels glabrous, leaf blades thick, SE USA	*Arundinaria*
–	Pedicels not glabrous, leaf blades thin, Himalayas & W China	*Sarocalamus*
4	Branches 3, all sheaths developed, basal nodes with thorns	*Chimonocalamus*
–	Branches 3-15, sheathing reduced, basal culm nodes with or without thorns	5
5	Rhizomes long or variable in length, clumps open or spreading	6
–	Rhizomes consistently short, culms in single clumps	7
6	Nodes raised, basal culm nodes usually with thorns, Africa	*Oldeania*
–	Nodes not raised, basal culm nodes without thorns, Asia	*Yushania*
7	Branch sheathing complete	*Thamnocalamus*
–	Branch sheathing reduced	8
8	Synflorescence branching paniculate	*Borinda*
–	Synflorescence branching racemose	9
9	Racemes unilateral, W China	*Fargesia*
–	Racemes not unilateral, Africa	*Bergbambos*

Sufficient data is now available to test whether this classification would gain support from molecular phylogenetic evidence. These two African species were not clearly resolved with Asian representatives of any genera in any molecular studies. For example, in a comparison of ITS sequences (Guo et al. 2002), *Thamnocalamus tessellatus* did not resolve with the type species of *Thamnocalamus*, *Thamnocalamus spathiflorus*, and its position varied between topologies. In the nuclear ribosomal ITS analysis of Hodkinson et al. (2010), *Yushania alpina* did not group with other *Yushania* species or closely with any other taxon. Weak associations between *Yushania alpina* and *Chimonocalamus* species were found by Guo and Li (2004) and Triplett (2008), which is interesting as they share possession of aerial roots developed into thorn-like structures, although they differ in other ways. However, neither *Yushania alpina* nor *Thamnocalamus tessellatus* resolved with putative relatives in these or similar genera of temperate bamboos in the most comprehensive studies undertaken so far, using sequences from 8 regions of cpDNA in 146 species and 26 genera (Zeng et al. 2010), and 108 bamboos from 25 genera using plastid DNA and nuclear GBSSI gene sequences (Zhang et al. 2012).

The molecular data would suggest that their inclusion in Asian genera would render those genera polyphyletic. Because their monotypic status is considered likely they could not be supported as monophyletic groups themselves in a classification based solely on molecular phylogeny. However, Zeng et al. (2010) and Zhang et al. (2012) considered them both to represent distinct lineages, and it is not possible to place them in well supported meaningful monophyletic groups except the tribe *Arundinarieae*. Therefore while the molecular data would not allow the diagnosis of monophyletic genera for the African bamboos following a strict Hennigian cladistic analysis, neither their current placement in *Thamnocalamus* and *Yushania*, nor placement in any other existing genus receives any support either. Continuing to include these bamboos in Asian genera causes serious problems when describing or distinguishing between those genera.

Although woody bamboos are considered to have evolved originally in Gondwanaland rather than Eastern Asia (Hodkinson et al. 2010), these African representatives are nested within the northern temperate clade, the tribe *Arundinarieae*, with a largely Asian distribution. This is estimated to have diverged from other woody bamboos around 23 mya (Hodkinson et al. 2010), 29 mya (Bouchenak-Khelladi et al. 2010) or 37.5 mya (Christin et al. 2008), but to have radiated only ca. 9 mya (Bouchenak-Khelladi et al. 2010) 10 mya (Hodkinson et al. 2010), or 19 mya (Christin et al. 2008). Peng et al. (2013) after sequencing 95% of the *Phyllostachys edulis* genome found evidence of whole genome duplication 7–12 mya, supporting the more recent dates.

Collision of tectonic plates has been suggested as a likely cause of this rapid radiation (Stapleton et al. 2009, Hodkinson et al. 2010). African and Indian plates met the Eurasian plate around that time, allowing a biotic interchange and subsequent radiation and diversification of Gondwanan elements into a wealth of new habitats. However, the temperate ancestors of these two African bamboo genera seem to have diverged around the same time that temperate bamboos arrived in Eastern Asia. Inclusion of endemic temperate bamboos from S India, Sri Lanka and Madagascar in a molecular phylogeny is required before any conclusions can be drawn as to where bamboos from the northern temperate clade first evolved, but there seems no evidence for an African origin, and it seems more likely that temperate bamboos radiated from India to Asia, Africa, and N America.

## Nomenclature

### Bergbambos

Stapleton gen. nov.

urn:lsid:ipni.org:names:77131102-1

http://species-id.net/wiki/Bergbambos

#### Remarks.

Differing from *Arundinaria* and *Sarocalamus* and similar to *Thamnocalamus* and *Fargesia* in its short-necked pachymorph rather than leptomorph rhizomes, and its compressed synflorescences. Differing from *Borinda* and *Thamnocalamus* in its racemose rather than paniculate synflorescence branching. Differing from *Fargesia* in the distichous rather than unilateral arrangement of spikelets in the racemes, the spikelets usually having only one fertile floret, and the scabrous pedicels. Differing from *Thamnocalamus* in the branch complement with reduced sheathing, and from *Fargesia* in the more varied orientation of the leaf blades.

#### Type.

*Bergbambos tessellata* (Nees) Stapleton comb. nov. urn:lsid:ipni.org:names:77131104-1 Basionym: *Nastus tessellatus* Nees, Fl. Afr. Austr. 1: 463. 1841. *Arundinaria tessellata* (Nees) Munro; *Thamnocalamus tessellatus* (Nees) Soderstrom & R.P. Ellis. Type: S Africa, Katberg, 4000–5000ft, J.F. Drège s.n. (lectotype, designated in Soderstrom & Ellis 1982, pg. 54: K!, http://apps.kew.org/herbcat/getImage.do?imageBarcode=K000345516

Rhizome pachymorph, short-necked, giving dense clumps. Culms to 7 m tall, diam. to 2 cm, nodding to pendulous, terete, smooth, nodes not raised and unarmed. Mid-culm branch complement initially with 5–7 main branches, erect, sheathing reduced. Culm sheaths persistent, tough. Leaf sheaths several to many, persistent, blades thick with random orientation. Synflorescence semelauctant, racemose, branch sheathing occasionally a soft sheath remnant, usually absent. Racemes not unilateral. Spikelets shortly pedicellate with 1(–2) fertile florets, pedicel scabrous. Empty glumes 2, no bud remnants. Lemma and palea similar in length. Stamens 3, filaments free. Stigmas 3. Lodicules 3.

Name *Bergbambos* from the Afrikaans name (Bergbamboes) in South Africa.

This genus would appear to be monotypic, confined to the mountains of South Africa, Lesotho and Swaziland.

### Oldeania

Stapleton gen. nov.

urn:lsid:ipni.org:names:77131103-1

http://species-id.net/wiki/Oldeania

#### Remarks.

Differing from *Arundinaria* and *Sarocalamus* and similar to *Yushania* in its long-necked pachymorph rather than leptomorph rhizomes, though similar to all in its open panicles. Differing from *Yushania* in its sulcate culm internodes, fewer, more horizontal branches, culm nodes with well developed supra-nodal ridge and often thorn-like aerial roots. Similar to *Chimonocalamus* in its panicles and thorn-like roots at culm nodes, but differing in its multiple branches with reduced sheathing and sulcate culm internodes.

#### Type.

*Oldeania alpina* (K. Schum.) Stapleton comb. nov. urn:lsid:ipni.org:names:77131105-1 Basionym *Arundinaria alpina* K. Schum. in Engler, Pflanzenwelt Ost-Afrikas 5: 117. 1895. *Sinarundinaria alpina* (K. Schum.) C.S. Chao & Renvoize; *Yushania alpina* (K. Schum.) W.C. Lin. Type: Kenya, Kikiju, G.A. Fischer 672 (holotype: B n.v., destroyed).

Rhizome pachymorph, long-necked, giving open stands and solitary culms. Culms to 15(–20) m tall, diam. to 6(–10) cm, erect to nodding, terete with shallow sulcus above branches, smooth, nodes with prominent supranodal ridge, in lower to mid culm a nodal ring of dense, short, hard, and thorn-like aerial roots often well developed. Mid-culm branch complement initially with 3–5 main branches, spreading, sheathing reduced. Culm sheaths deciduous, tough. Leaf sheaths several to very many, blades thick. Synflorescence semelauctant, paniculate, branch sheathing reduced to hard bracts, soft sheath remnants or hairs. Spikelets pedicellate with several fertile florets, pedicel scabrous. Empty glumes 2, bud remnants present or absent, fertile glumes 4–8. Lemma and palea similar in length. Stamens 3, filaments free. Stigmas 2. Lodicules 3.

Name *Oldeania* from the Maasai common name (Oldeani) in Tanzania.

Currently only the type species can be reliably placed in the genus, which thus has a distribution across tropical Africa from Cameroon in the west to E Africa, where it occurs from Ethiopia south to Tanzania. There is a possibility that species from Madagascar will be placed in this genus when they are better known, but they may be conspecific or even introduced. It provides important montane wildlife habitats and food, notably for the critically endangered Mountain Gorilla, *Gorilla beringei beringei*.

The holotype, G.A. Fischer 672, was destroyed by fire during the 1939–1945 World War. No trace of the type collection or any duplicate has been found in surviving components of the Berlin collections, nor in other herbaria, including the Hamburg collections taken to Russia and recently repatriated (Poppendieck pers. comm.). The likelihood of substantial infraspecific variation, the possibility of further species, and the lack of other collections from the type locality together make it inadvisable to select a neotype or epitype until new collections have been made.

## Supplementary Material

XML Treatment for Bergbambos

XML Treatment for Oldeania
